# Strategies to Alleviate the Burden Experienced by Informal Caregivers of Persons With Severe Mental Disorders in Low- and Middle-Income Countries: Scoping Review

**DOI:** 10.2196/48587

**Published:** 2024-01-18

**Authors:** Olindah Silaule, Daleen Casteleijn, Fasloen Adams, Nokuthula Gloria Nkosi

**Affiliations:** 1 Department of Occupational Therapy University of the Witwatersrand Johannesburg South Africa; 2 Department of Occupational Therapy University of Pretoria Pretoria South Africa; 3 Division of Occupational Therapy Stellenbosch University Cape Town South Africa; 4 Department of Nursing Education, University of the Witwatersrand Johannesburg South Africa

**Keywords:** severe mental disorders, informal caregivers, caregiver stress, caregiver support, low- and middle-income country, mobile phone

## Abstract

**Background:**

There is considerable evidence of the burden of care encountered by informal caregivers of persons with severe and enduring mental health conditions in low- and middle-income countries. Previous studies have highlighted the need to support these informal caregivers as key players in the care of these patients. To date, limited evidence exists on the extent and types of strategies for supporting these informal caregivers in low- and middle-income countries.

**Objective:**

This scoping review aims to identify and describe the extent and type of evidence on the existing strategies for alleviating the burden of care among informal caregivers of persons with severe and enduring mental health conditions in low- and middle-income countries.

**Methods:**

A systematic literature search was completed following the Joanna Briggs Institute methodology for scoping reviews. The participants, concept, and context framework was used to guide the search for literature sources across 5 databases: PubMed, MEDLINE, CINAHL, and PsycINFO for published literature and ProQuest for unpublished literature. This review included studies that reported on strategies for alleviating the burden of care among informal caregivers of persons with severe and enduring mental health conditions, with a focus on studies that evaluated or recommended caregiver interventions and support strategies in low- and middle-income countries. The search was limited to studies conducted between 2001 and 2021, and only papers written in English were considered for inclusion. Using the Covidence software (Veritas Health Innovation), 2 reviewers independently screened the papers, applied the inclusion and exclusion criteria, and met biweekly to discuss and resolve conflicts. The relevant studies and reported outcomes were summarized, organized, and analyzed descriptively using numeric summary analysis and deductive content analysis.

**Results:**

Of the 18,342 studies identified, 44 (0.24%) met the inclusion criteria. The included studies were from 16 low- and middle-income countries in Asia, Africa, Europe, and South and North America. Most studies (21/44, 48%) were randomized controlled trials conducted in Asian countries. The identified strategies were grouped into 2 categories: implemented and recommended intervention strategies. Identified strategies included community-based interventions, psychoeducation interventions, support groups, cognitive behavioral therapy, spirituality-based interventions, and smartphone-based interventions. In addition, mindfulness and empowerment, collaborative interventions, standard care, financial and social support, counseling, occupation-based interventions, policy and legislature, and access to mental health care were identified. Psychoeducation and support group interventions were identified as common strategies for alleviating the burden of care among informal caregivers of persons with severe and enduring mental health conditions.

**Conclusions:**

This review provides evidence on the types of implemented and recommended strategies for alleviating the burden of care among informal caregivers in low- and middle-income countries. Although psychoeducational interventions were the most preferred strategy for alleviating burden, their benefits were short-lived when compared with peer-led support groups.

**International Registered Report Identifier (IRRID):**

RR2-10.2196/44268

## Introduction

### Background Functional Implications of Severe and Enduring Mental Health Conditions

People with severe and enduring mental health conditions are unable to cope with the demands of everyday life and need care after being discharged from a health facility. Serious functional limitations frequently interfere with the ability of people with severe and enduring mental health conditions to perform essential roles such as being a worker, family member, or friend [1]. The lack of independence among people with severe and enduring mental health conditions in daily living activities and, in some cases, behavioral problems result in poor quality of life. Psychosis, bipolar mood, major depressive, anxiety, eating, and personality disorders are often classified as severe and enduring mental health conditions [2]. The amount of care needed varies greatly depending on the diagnosis, severity of symptoms, and level of independent functioning. As such, assistance is needed in areas of basic self-care, healthy eating, following daily routines, medication management and compliance, engagement in meaningful activities, and community integration. Therefore, informal caregivers are essential to fulfill the care needs of people with severe and enduring mental health conditions. This task becomes draining, and the caregiver burden should not be underestimated [3,4].

### Informal Caregivers in Mental Health

Informal caregivers are people who deliver care without remuneration to persons with a chronic condition. The caregivers of persons with severe and enduring mental health conditions are most often family members or neighbors who assist with the care of the person with a mental health condition [5]. Dixon et al [6] noted that, often, the caregiver of a person with a severe and enduring mental health condition does not have a choice in being the caregiver, which is an additional burden of care for the carer. Informal caregivers’ roles include monitoring medication, being the contact person between the health provider and family, early identification of signs of relapse, taking care of daily tasks such as self-care, providing meals, and ensuring the safety of the person [7,8]. Informal caregivers provide emotional support when needed and deal with the challenging behavior of the person with a severe and enduring mental health condition, which may lead to police involvement [9]. Involving informal caregivers in the routine care and management of persons with severe and enduring mental health conditions has shown a positive influence on the course of the illness but only if the caregivers’ needs are addressed and they are supported in one way or another [5,6,10].

### Caregiver Burden and Need for Support

The burden of care experienced by caregivers is classified into objective and subjective burden. Objective burden refers to the tangible impact of the demands of caring tasks and encompasses the practical and concrete aspects of caregiving that can be quantified or assessed externally [11,12]. Subjective burden refers to the emotional and psychological experiences, feelings, and perceptions of caregivers related to their caregiving role [8,13]. It focuses on how caregivers perceive the impact of their responsibilities on their well-being and mental health.

In low- and middle-income countries, objective burden can easily overshadow subjective burden, such as a lack of community mental health care services and clinics that are out of stock of medication for people with severe and enduring mental health conditions, causing relapse and often readmission [11]. Other concrete aspects of objective burden, such as long hours of being available to the care recipient, time lost from daily activities, and not being able to earn an income, have serious consequences for caregivers as they are unable to pursue their own goals in life and, accordingly, experience a lower quality of life [9,12]. Cultural beliefs about being cursed by ancestors [14] and stigma from health care professionals [15] further aggravate the caring duties of caregivers, precipitating objective burden. The objective burden of care is less reported in the literature than subjective feelings of burden. Many studies have reported the subjective burden of care, which highlights emotional distress and feelings of anxiety, depression, and sadness related to the challenges of caregiving and witnessing the struggles of their family member. Loss of freedom and autonomy has also been reported owing to the demand of caregiving [16]. Feelings of shame and social isolation stem from the stigma of mental illness and cultural beliefs about being cursed by ancestors. Caregivers can also experience role strain as they have to juggle responsibilities at home, which can cause fatigue and overall dissatisfaction in life [17]. Both dimensions of burden are crucial for understanding the challenges faced by family caregivers of individuals with severe and enduring mental health conditions and play a vital role in informing support strategies and interventions.

There is overwhelming evidence in the literature for the need to support caregivers, and several studies from high-income and low- and middle-income countries have indicated successful strategies for alleviating caregiver burden. Studies on informal caregivers of people with severe and enduring mental health conditions in low- and middle-income countries have also increased over the past 10 years, and the burden of caregivers has been well described [18-21]. Yerriah et al [16] reported on the extent of the burden of caregivers of persons with schizophrenia in rural South Africa and found that this sample has a higher burden of care. A recommendation from this study was to develop strategies to support caregivers with the aim of improving their quality of life.

### Support Strategies for Caregivers

To date, a number of strategies to support caregivers have been investigated with varying results. In Turkey, family-to-family support programs have shown a positive impact on the burden of care [22]. A meta-analysis by Chen et al [23] showed some support for nonpharmacological interventions (mostly psychoeducation) for caregivers of persons with schizophrenia, but the authors reported a potential bias in the results because of the small sample size. In contrast, the systematic review and meta-analysis by Sin et al [24] did not support psychoeducation to improve compliance with treatment and prevent relapse in persons with psychosis, and they reported a lack of available data; thus, no meta-regressions could be conducted. Ewertzon and Hanson [18] conducted a narrative review and identified provision of knowledge, problem-solving, stress management, mutual support groups, and individual-support interventions as successful support interventions for caregivers. Finally, a systematic review by Napa et al [25] revealed that there is insufficient evidence of interventions for psychological distress and expressed emotions in families of persons who experienced first-episode psychosis.

There is limited evidence of web-based health care services and digital health technologies for supporting informal caregivers of individuals with severe and enduring mental health conditions [26]. Sin et al [27] developed an eHealth intervention called Carers for People with Psychosis e-support, but its effectiveness has not yet been investigated. Ploeg et al [28] conducted a rapid review of web-based interventions to improve general caregiver outcomes. More than half of the 17 included studies showed a positive outcome for decreased depressive symptoms, stress, and anxiety among caregivers. If virtual strategies can support caregivers in rural areas, they may be a feasible method to reach people in remote areas who have poor access to health care.

Informal caregivers in rural or remote areas face additional objective burdens such as poor access to services, lack of integration of mental health into community health services [29], and poor intersectoral collaboration. They often have to wait long hours before any support arrives, and in many cases, they have to deal with challenges with the limited resources they have available.

If the types of intervention strategies that could be relevant for low- and middle-income countries, how they were implemented, and the outcome that was achieved were mapped, it could guide health care workers to support informal caregivers on various levels and with various strategies. The availability of virtual support strategies could add another dimension of support to caregivers, which may lead to additional positive outcomes such as immediate support, available information, and contact with support groups. People with severe and enduring mental health conditions benefit as they are most likely to receive optimal care, and their relapse rate may decrease, which means less need for readmission in overextended mental health care wards or hospitals. In addition, people with severe and enduring mental health conditions may also experience a better quality of life if their carers are supported. Thus, it is essential to understand the strategies to alleviate informal caregiver burden and how these strategies should be implemented.

This scoping review aimed to map the strategies to alleviate the objective and subjective burden of informal caregivers of persons with severe and enduring mental health conditions in low- and middle-income countries. The objectives of this scoping review were to (1) identify the types of existing strategies (virtual and face-to-face) for alleviating the objective or subjective burden of care, (2) describe the characteristics of the identified strategies, and (3) list the positive outcomes that were achieved using the identified strategies.

## Methods

### Review Methodology

This scoping review followed the Joanna Briggs Institute methodology specific to scoping reviews [30]. An a priori protocol for this review has been published [31].

### Review Question

The scoping review addressed two research questions related to the strategies for alleviating the burden of care among informal caregivers of persons with severe and enduring mental health conditions:

1. Which existing strategies are reported in the literature for alleviating the burden of informal caregivers of persons with severe and enduring mental health conditions in low- and middle-income countries?
2. What are the outcomes reported by the authors of the strategies for alleviating burden among informal caregivers?

### Eligibility Criteria

#### Participants

Studies were included if participants were informal caregivers of persons with severe and enduring mental health conditions in low- and middle-income countries. Studies that focused on informal caregivers of patients diagnosed with Alzheimer disease and dementia were excluded. This included family, friends, neighbors, and community members who voluntarily provided care without any remuneration. Studies that included both informal caregivers and patients as participants were considered for inclusion, whereas those that reported only on patients were excluded.

#### Concept

Caregiver burden was defined as the physical, psychological, emotional, social, and financial stresses of providing care to a person with severe and enduring mental health conditions [32]. Severe and enduring mental health conditions in this scoping review included the schizophrenia spectrum and other psychotic disorders, bipolar and related disorders, mood or depressive disorders, and personality disorders. Studies that investigated strategies for alleviating caregiver burden were considered for inclusion. This also included studies that investigated the synonyms used to describe the burden of care among informal caregivers, such as *caregiver strain*, *stress*, and *role fatigue*. Studies that reported on the burden of care without suggesting strategies for alleviating it were excluded.

#### Context

Studies from low- and middle-income countries, including countries in Africa, Asia, Latin America, and the Caribbean, were considered for inclusion. In addition, the inclusion criteria comprised studies from lower-middle-income countries using the World Bank classification of the economic status of a country at the time the study was conducted. The term *developing country* was included as it is similar to *lower-middle-income country*, and thus, countries in Africa, Asia, and Latin America (including the Caribbean) were eligible for inclusion.

#### Types of Sources

This review included published qualitative, quantitative, and mixed methods studies with all types of designs as well as unpublished studies, including dissertations and theses. Over the last decade, there has been a growing body of knowledge from studies outlining strategies for alleviating burden among informal caregivers in low- and middle-income countries. As a result, this review was only restricted to studies conducted between 2011 and 2021. Only papers written in English were included in this review.

### Search Strategy

A 3-step search strategy process was used to identify relevant studies. The search strategy commenced with an initial limited search of PubMed, MEDLINE, CINAHL, and PsycINFO for published studies between 2011 and 2021. The keywords were as follows: “[informal caregiver/s OR caregiver/s] AND [caregiver burden OR caregiver stress] AND [support strategy/ies OR intervention/s] AND [severe mental disorder/s OR mental illness] AND [developing country/ies].”

The second search was refined with the assistance of the librarian at the University of the Witwatersrand, and additional terms were added: “[Carers or ‘Informal Carers’ or ‘Male Caregiver’ or Women Caregiver’ or ‘Family Caregivers’] AND [Caregiver* strain’ or ‘Caregiver *Exhaustion’ or ‘Caregiver* burnout’ or ‘Carer* burden’] AND [‘underserved’ or ‘deprived’ or ‘middle-income’ or low income country].” On advice from the librarian, gray literature such as conference presentations and unpublished studies was searched on ProQuest Dissertations and Theses Global and public health and conference databases. Finally, 2 reviewers (OS and DC) screened the reference lists of the included articles for additional studies. Textbox 1 presents the final list of search terms used to search published and unpublished studies across all the selected databases in this review. The identified references were imported into the Mendeley reference manager (Elsevier) for screening.

**Search terms used in the databases.**

‘Informal caregiver*’ or Caregiver* or Carers or ‘Informal Carers’ or ‘Male Caregiver’ or ‘Women Caregiver’ or ‘Family Caregivers’ or ‘Unpaid Caregiver’ or ‘Spouse Caregiver’ or ‘Caretaker’ or ‘Older Caregiver’

AND

‘Caregiver* burden’ or ‘Caregiver* stress’ or ‘Caregiver* strain’ or ‘Caregiver *Exhaustion’ or ‘Caregiver* burnout’ or ‘Carer* burden’ or ‘Carer* stress’ or ‘Caretaker* role fatigue’ or ‘Burden of Caregiver*’ or ‘Caretaker* burden’ or ‘Caretaker* load’ or ‘Caregiver* Psychology’

AND

‘Strategy’ or ‘Strategies’ or ‘Intervention’ or ‘Procedure’ or ‘Programme*’ or ‘Management’ or ‘Protocol* Guidelines’ or ‘Guide’ or ‘Policy’ or ‘Policies’

AND

‘Mental disorders’ or ‘Mental illness’ or ‘schizophrenia’ or ‘bipolar mood disorder’ or ‘Major depressive disorder’ or ‘Psychotic disorder’ or ‘Personality disorder’ or ‘Bipolar affective disorder’

AND

(developing OR (less* N1 developed) OR “under developed” OR underdeveloped OR “under served” OR underserved OR deprived OR poor* OR “middle income” OR (low* N1 income)) N1 (countr* OR nation* OR population* OR world)) OR ((developing OR (less* N1 developed) OR “under developed” OR underdeveloped OR “under served” OR underserved OR deprived OR poor* OR “middle income” OR (low* N1 income)) N1 (countr* OR nation* or population* OR world)) OR ((developing OR (less* N1 developed) OR “under developed” OR underdeveloped

### Source of Evidence Selection

Following the search, all identified citations were exported to the Mendeley reference manager and thereafter to a web-based software, Covidence (Veritas Health Innovation), for primary screening and data extraction from the selected articles. Duplicates were removed, and 2 reviewers (OS and DC) independently conducted the screening for titles, abstracts, and full texts using the inclusion and exclusion criteria. To enhance the reliability of the review results, the first 5 abstracts were screened, and the 2 reviewers compared the screening results and clarified conflicts. Following this, the criteria were revised to ensure a detailed description of the various types of burden. The remaining abstracts were screened, and comparisons were made after 10 screenings to manage conflicts. The 2 reviewers (OS and DC) proceeded to screen texts and extract data from the selected articles. Any disagreements regarding the inclusion of the studies were resolved through discussion between the 2 reviewers**.**

### Data Extraction

**Figure 1:**
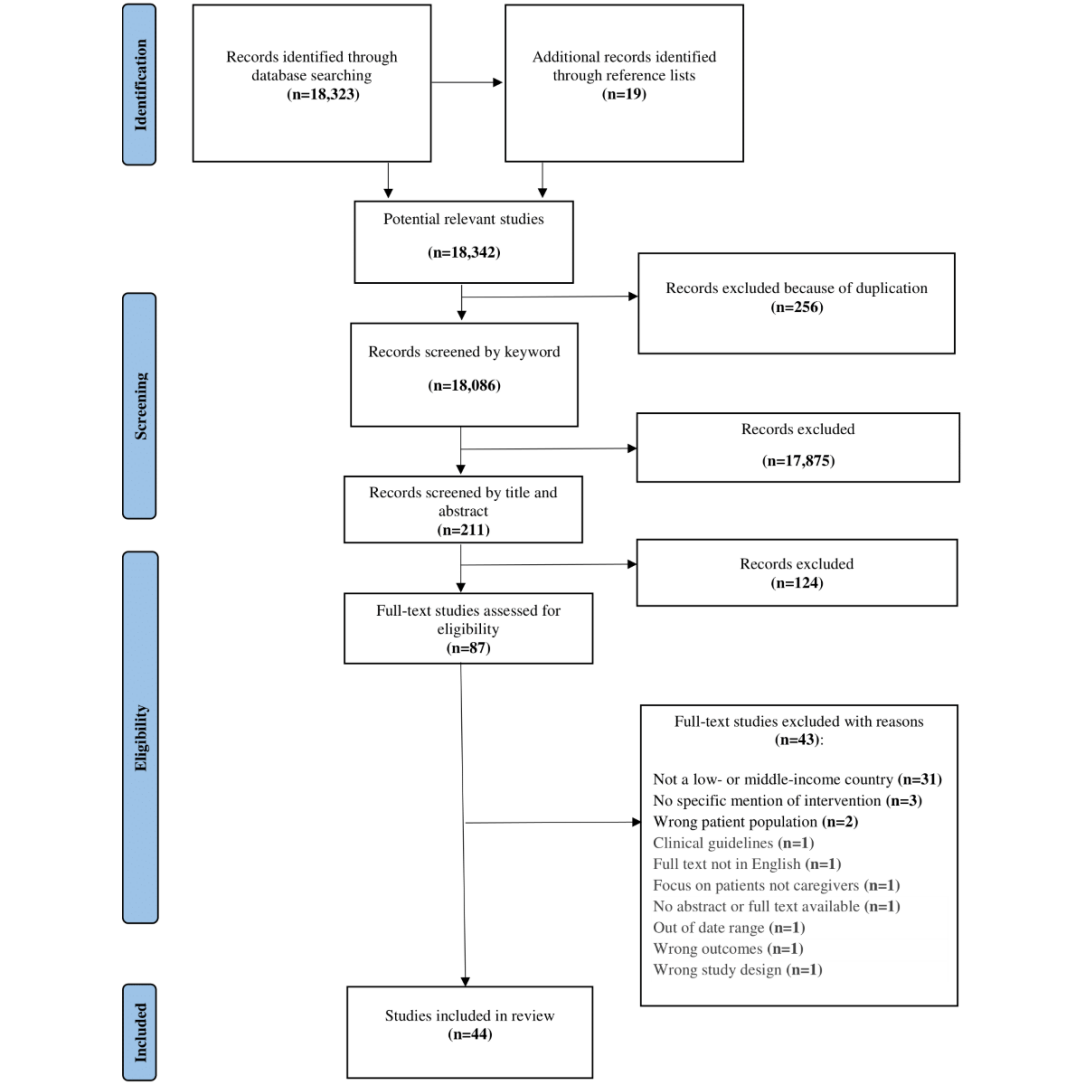
PRISMA (Preferred Reporting Items for Systematic Reviews and Meta-Analyses) flow diagram.

The Covidence software has templates for data extraction. These templates were modified using the data extraction tables proposed by Peters et al [30]. To ensure the extraction of relevant data to answer the review question, these templates were piloted by the reviewers before use. In total, 2 separate data extraction templates (Multimedia Appendix 1) were used to extract data from studies that implemented an intervention and from studies that recommended interventions and strategies for alleviating caregiver burden. Data related to the characteristics of the studies were extracted, including study title; study aims; citation details; population of interest; concept of interest; context of the study, including the country and type of setting; type of evidence sources; study approach and designs; and participant characteristics, such as age, gender, and diagnosis of the care recipients. In addition, information on the characteristics of the interventions was extracted. This included the intervention content (ie, type of intervention, intervention developer and deliverer, and type of burden targeted by the intervention). The intervention description included the duration of the intervention, number of sessions, and location for the intervention. The templates for extracting the data are available in Multimedia Appendix 1. The reviewers extracted the data independently. Web-based meetings were held biweekly to discuss and resolve any discrepancies in the extracted data. Regular comparisons were easy and quick to conduct and improved the consistency of the extracted data. After completing the data extraction, the 2 reviewers scanned the references of the included articles to ensure that no articles were missed. Included and excluded studies were reported in a PRISMA-ScR (Preferred Reporting Items for Systematic Reviews and Meta-Analyses extension for Scoping Reviews) flow diagram (Figure 1).

### Data Analysis and Presentation

To increase consistency in the data, this review followed a 3-step analysis process as proposed by Levac et al [33]. This included analyzing the data, reporting the results, and applying meaning to the results [33]. The first step was reviewing the extracted data and identifying the type of data to be analyzed, which were then grouped according to the objectives of the review. This was followed by identifying the type of analysis appropriate for the specific type of data and then analyzing the extracted data. A descriptive quantitative analysis was conducted in a Microsoft Excel (Microsoft Corp) spreadsheet to describe the characteristics of the studies. This included the overall number of studies, types of study design, years of publication, characteristics of the study populations, countries where the studies were conducted, and types of interventions. In addition, descriptive content analysis was conducted deductively using the NVivo software (Lumivero) to code the characteristics of the extracted data into overall categories [34]. Data were categorized into types of strategies—implemented and recommended intervention strategies—and the outcomes of the strategies aimed at alleviating the burden of informal caregivers as reported by the authors. To enhance the clarity of the emerging findings, the data were summarized and presented in graphs and tables.

## Results

### Study Inclusion

A total of 18,323 studies were identified from the databases using the keywords. In total, 19 additional studies were identified from the reference lists of the included studies, thereby bringing the total number of potentially relevant studies to 18,342. Subsequently, of the 18,342 studies, 256 (1.4%) duplicates were removed, thereby leaving 18,086 (98.6%) studies deemed relevant to the review based on the keywords. A total of 98.83% (17,875/18,086) of these studies were then excluded; this exclusion of numerous studies occurred because of their incongruence with the predefined inclusion criteria, such as instances in which the study did not pertain to a low- or middle-income country context or in which the primary emphasis lay on the patient rather than the caregiver. Therefore, 1.17% (211/18,086) of the studies were screened by title and abstract based on set inclusion and exclusion criteria. This led to the exclusion of 58.8% (124/211) of these studies from the review. A total of 41.2% (87/211) of the studies were then retrieved as full texts based on eligibility by screening their titles and abstracts. Of these 87 studies, 43 (49%) were excluded for various reasons: 31 (72%) were not from low- or middle-income countries; 3 (7%) did not specify the intervention strategy; 2 (5%) focused on the wrong patient population; and another 7 (16%) were excluded for being a clinical guideline, focusing on patients rather than caregivers, having misaligned outcomes and a study design within the exclusion criteria (eg, review articles), being out of the date range of this review, the full text being in a language other than English, and a lack of an abstract and full text. Finally, 44 studies were included in this scoping review. The PRISMA (Preferred Reporting Items for Systematic Reviews and Meta-Analyses) flow diagram was used to present the selection of the studies (Figure 1). It took approximately 8 months, from September 2021 to June 2022, to carry out the study from conception to completion of the project.

### Characteristics of the Eligible Studies

#### Summary of Studies and Evidence Type

The included studies were published between 2011 and 2021. Most studies (31/44, 70%) were published between 2016 and 2021, and 30% (13/44) were published between 2011 and 2016. The types of studies included peer-reviewed journal articles (40/44, 91%), research reports or theses (2/44, 5%), and opinion pieces (2/44, 5%). Of the 44 included studies, 29 (66%) reported on a specific implemented intervention strategy, and 15 (34%) outlined recommended intervention strategies aimed at alleviating the burden of informal caregivers of people with severe and enduring mental health conditions in low- and middle-income countries. The study designs included randomized controlled trials (21/44, 48%), quantitative studies (15/44, 34%), qualitative studies (3/44, 7%), mixed methods studies (3/44, 7%), and gray literature (2/44, 5%; Table 1).

**Table 1:** Characteristics of the included studies (N=44).

Characteristic		Studies, n (%)
**Summary of studies**		
	Implemented strategy studies	29 (66)
	Recommended strategy studies	15 (34)
**Type of evidence**		
	Journal articles	40 (91)
	Research reports or theses	2 (5)
	Opinion pieces	2 (5)
**Year of publication**		
	2011-2015	13 (30)
	2016-2021	31 (70)
**Study design**		
	Randomized controlled trial	21 (48)
	Quantitative design	15 (34)
	Qualitative design	3 (7)
	Mixed methods	3 (7)
	Gray literature	2 (5)

#### Context of the Studies

A total of 44 studies identified from 16 low- and middle-income countries in Asia, Africa, Europe, and South and North America (Mexico) were included. The countries included China (12/44, 27%); Iran (10/44, 23%); India (5/44, 11%); Brazil (3/44, 7%); Greece (2/44, 5%); Ghana (2/44, 5%); and Thailand, Nepal, South Korea, Indonesia, Jordan, Mexico, Ethiopia, Turkey, Botswana, and mixed countries (including Brazil, Iran, Colombia and Mexico 1/44, 2% of the studies each). As shown in Table 2, majority of the studies with implemented strategies (24/29, 83%) and those with recommended strategies (9/15, 60%) were from Asian countries. Overall, 7% (2/29) of the studies each from Europe, South America, and North America implemented the strategies for alleviating burden and 13% (2/15) of the studies from South and North America recommended the strategies. Only 3% (1/29) of the studies conducted in Africa implemented the strategies and 20% (3/15) of the studies recommended the strategies. A study (1/15, 7%) from mixed countries recommended the strategies for alleviating burden.

**Table 2:** Continents of the recommended and implemented strategies (N=44).

Continent	Implemented strategies, n (%)	Recommended strategies, n (%)
Asia	24 (83)	9 (60)
Africa	1 (3)	3 (20)
Europe	2 (7)	0 (0)
South and North America	2 (7)	2 (13)
Mixed countries	0 (0)	1 (7)

#### Characteristics of the Study Populations

The targeted populations in the included studies were mainly family caregivers (38/44, 86%), 7% (3/44) of the studies targeted informal carers, and some studies (3/44, 7%) did not specify their targeted population but rather identified them as caregivers. Most studies (33/44, 75%) included both male and female caregivers, and 25% (11/44) did not specify the sex of their sample. Similarly, most studies (24/44, 55%) included both male and female care recipients, only 2% (1/44) of the studies focused only on male care recipients, and 43% (19/44) did not specify the gender of the care recipients included in the studies. The diagnoses of the care recipients in the included studies were grouped into 6 categories: schizophrenia (17/44, 39%), mood disorders (4/44, 9%), multiple diagnoses including schizophrenia and mood disorders (5/44, 11%), mixed severe and enduring mental health conditions (5/44, 11%), other psychotic and chronic conditions (6/44, 14%), and unspecified mental disorders (7/44, 16%; Table 3).

**Table 3:** Characteristics of the study populations in the included studies (N=44).

Characteristic		Studies, n (%)
**Population of interest**		
	Family caregiver or caregivers	38 (86)
	Informal carer or carers	3 (7)
	Caregiver or caregivers	2 (5)
	Carer or carers	1 (2)
**Concept of interest**		
	Caregiver burden	18 (41)
	Family burden	16 (36)
	Caregiver stress	9 (20)
	Caregiver strain	1 (2)
**Sex of caregivers**		
	Mixed-gender groups (male and female)	33 (75)
	Not specified	11 (25)
**Gender of care recipients**		
	Mixed-gender groups (male and female)	24 (55)
	Male	1 (2)
	Not specified	19 (43)
**Diagnosis of care recipients**		
	Schizophrenia	17 (39)
	Mood disorders	4 (9)
	Schizophrenia and mood disorders	5 (11)
	Mixed diagnoses of severe mental disorders	5 (11)
	Unspecified mental disorders	7 (16)
	Other (drug dependency, psychotic disorders, and chronic conditions)	6 (14)

### Concept of Interest

The concepts of interest reported in the studies included caregiver burden (18/44, 41%), family burden (16/44, 36%), caregiver stress (9/44, 20%), and caregiver strain (1/44, 2%; Table 3).

### Review Findings

This section outlines the findings of the scoping review and includes the types of intervention strategies, characteristics of the intervention strategies, and author-reported outcomes from the included studies.

#### Types of Intervention Strategies

The various strategies for alleviating caregiver burden were grouped into 2 categories: implemented and recommended intervention strategies. The implemented intervention strategies emerged from studies that investigated the effects of a specific intervention to alleviate the burden of informal caregivers. The recommended interventions were identified from studies that investigated caregiver burden and outlined recommended intervention strategies to be used to alleviate the burden of informal caregivers of persons with severe and enduring mental health conditions. Most studies (29/44, 66%) reported on an implemented intervention strategy, and 34% (15/44) of the studies recommended strategies to alleviate caregiver burden.

Most of the studies and articles (33/44, 75%) originated in Asia, with 55% (24/44) implemented and 20% (9/44) recommended intervention strategies reported on, followed by Africa with 2% (1/44) implemented and 7% (3/44) recommended intervention strategies, South and North America with 5% (2/44) implemented and 5% (2/44) recommended intervention strategies, and Europe with 5% (2/44) implemented intervention strategies (Table 2). A study from mixed countries only reported recommended intervention strategies. The identified strategies were grouped into the following categories: community-based interventions, psychoeducation interventions, support groups, cognitive behavioral therapy (CBT), spirituality-based interventions, smartphone-based interventions, mindfulness and empowerment, collaborative interventions, standard care, financial and social support, counseling, occupation-based interventions, policy and legislature, and access to mental health care (Table 4 and Textbox 2). These categories are discussed in the following subsections.

**Table 4:** Description of the identified evaluated strategies for alleviating informal caregiver burden.

Type of strategy	Evaluated interventions	Author-reported outcomes
Community-based interventions	CoMHIP^a^ RESHAPE^b^	Positive outcomes (positive effects on family caregivers)
Psychoeducation interventions	Psychoeducation program Group psychoeducation FLEP^c^ Peer-assisted education	Improved coping skills Enhanced recovery Improved QoL^d^ Knowledge and skill acquisition Reduced burden of care Reduced anxiety and stress Reduced psychological strain Enhanced family functioning Relapse prevention Reduced prolonged admissions Negative outcomes (no efficacy on burden of care, QoL, or self-esteem among BMD^e^ caregivers)
Support groups	FPGP^f^ Family-led mutual support program	Knowledge and skill acquisition Enhance help seeking Alleviation of guilt Improved family and patient functioning Decreased demand on mental health services Improved psychosocial health Improved QoL
Cognitive behavioral therapy	Emotional regulation training	Reduced burden of care Reduced anxiety and stress Increased resilience
Spirituality-based intervention	Spirituality-based program	Reduced anxiety and stress
Guided self-help interventions	Manual-guided PBSP^g^ The Good Mood Guide	Strengthened positive caregiving experience Reduced negative caregiving experience Increased access to support
Smartphone-based interventions	MHapps^h^	None
Mindfulness and empowerment interventions	IEP^i^ MBSR^j^ program	Caregiver empowerment Improved QoL Decreased depressive symptoms Increased self-efficacy
Collaborative interventions	Participatory care model	Reduced burden of care Increased resilience
Standard care	Multimodal intervention (general medicine, psychiatry, psychology, family therapy, neuropsychological rehabilitation, and occupational therapy)	Reduced burden of care Increased social support

^a^CoMHIP: community mental health early intervention project.

^b^RESHAPE: Reducing Stigma Among Healthcare Providers.

^c^FLEP: Family Link Education Programme.

^d^QoL: quality of life.

^e^BMD: bipolar mood disorder.

^f^FPGP: Family-led Peer Support Group Program.

^g^PBSP: problem-solving–based self-learning program.

^h^MHapps: mental health apps.

^i^IEP: integrated empowerment program.

^j^MBSR: mindfulness-based stress reduction.

**Description of the identified recommended strategies for alleviating informal caregiver burden.**

**Community-based interventions**

- Strengthening existing primary health care system
- Establishment of day nursing or care
- Respite care
- Vocational training for patients
- Single-family caregiver organization
- Supported employment for patients
- Home visits

**Psychoeducation interventions**

- Group psychoeducation
- Family education
- Continuing education programs for effective practice
- Educational lectures
- Psychoeducation program

**Support groups**

- Nongovernmental mental health–related support groups
- Family caregiver assistance programs

**Cognitive behavioral therapy**

- Family therapy groups

**Spirituality-based intervention**

- Turning to traditional healers and spiritual leaders

**Smartphone-based interventions**

- Mental health apps

**Mindfulness and empowerment interventions**

- Stress management training

**Collaborative interventions**

- Caregiver involvement in all program elements
- Pluralistic and ecological approach to service delivery

**Standard care**

- Telepsychiatry
- Availability of emergency teams

**Financial support**

- Disability grant
- Fee-free mental health services
- Medical insurance and free medication

**Social support**

- Medical care social security
- Social resources

**Counseling**

- Basic counseling
- Supportive psychotherapy

**Occupation-based interventions**

- Physical and leisure activities

**Policy and legislature**

- National health insurance schemes
- Advocacy for a strong mental health policy
- Integration of caregiver actions and interventions into national mental health care plans
- Fee-free mental health services

**Access to mental health care**

- Caregiver care inclusion in daily treatment facilities
- Access to therapeutic tools
- Availability of mental health units or departments
- Periodic health checks for caregivers (every 6 months on average)
- Improvement in institutional mental health care

#### Psychoeducational Interventions

The first strategy was psychoeducational interventions, which was identified as the most common strategy implemented and recommended to alleviate caregiver burden. A total of 27% (12/44) of the studies implemented 4 different psychoeducational interventions, including 58% (7/12) of these studies using a psychoeducation program [35-40], 17% (2/12) using group psychoeducation [41,42], 17% (2/12) using the Family Link Education Programme [43,44], and 8% (1/12) of the studies implementing peer-assisted education [45] (Table 4). Similarly to the implemented intervention strategies, the recommended psychoeducational interventions identified included psychoeducational programs, group psychoeducation, family psychoeducation, continuing education programs for effective practice, and educational lectures [7,46] (Textbox 2).

#### Support Group Interventions

The second strategy was support groups. In total, 7% (3/44) of the studies implemented support group strategies, including the Family-led Peer Support Group Program, family-led mutual support programs, and nongovernmental mental health–related support groups [47-49] (Table 4). The recommended support group strategies identified in this review included nongovernmental mental health–related support groups and family caregiver support programs [50-54] (Textbox 2).

#### Community-Based Interventions

Community-based interventions were identified as a third strategy to alleviate caregiver burden, and 5% (2/44) of the included studies reported on implementing a community mental health early intervention project and Reducing Stigma Among Healthcare Providers [55,56] as community-based interventions (Table 4). The recommended community-based interventions included strengthening existing primary health care systems, establishing day nursing or care for the care recipients, respite care, vocational rehabilitation training for patients, single-family caregiver organizations, supported employment for patients, and home visits [46,52,53,57] (Textbox 2).

#### Guided Self-Help Interventions

A total of 5% (2/44) of the studies implemented guided self-help interventions, namely, a manual-guided problem-solving–based self-learning program [58] and the Good Mood Guide [59] (Table 4). No recommended guided self-help interventions were identified in this review.

#### Mindfulness and Empowerment Interventions

Mindfulness and empowerment interventions were implemented in 5% (2/44) of the studies, and the programs implemented were a mindfulness-based stress reduction program and an integrated empowerment program [60,61] (Table 4). Stress management training was identified as a recommended mindfulness and empowerment intervention strategy in this review [7] (Textbox 2).

#### CBT Interventions

Emotional regulation training was the only implemented CBT strategy identified in 5% (2/44) of the included studies [62,63] (Table 4). The recommended cognitive behavioral strategy identified in this review was family therapy groups [64] (Textbox 2).

#### Spirituality-Based Interventions

In total, 2% (1/44) of the studies implemented a spirituality-based program for informal caregivers in which the comparison intervention was 2 standard group training sessions related to general mental disorders [65] (Table 4)*.* Turning to traditional healers and spiritual leaders was identified as a recommended spirituality-based intervention for alleviating informal caregiver burden [52] (Textbox 2).

#### Participatory Care Model

In total, 2% (1/44) of the studies implemented a participatory care model as a collaborative intervention strategy [66] (Table 4). The recommended collaborative interventions were caregiver involvement in all program elements and a pluralistic and ecological approach to service delivery [56,67] (Textbox 2).

#### Standard Care

The implemented standard care interventions were multimodal interventions that encompassed general medicine, psychiatry, psychology, family therapy, neuropsychological rehabilitation, and occupational therapy with no comparison intervention [68] (Table 4). The recommended standard care interventions were telepsychiatry and ensuring the availability of emergency teams [53] (Textbox 2).

#### Smartphone-Based Interventions

Only 2% (1/44) of the studies implemented smartphone-based interventions using mental health apps to alleviate the burden of informal caregivers with no comparison intervention [67] (Table 4). Although the study indicated the use of the mental health apps with care recipients and their caregivers, the specific intervention offered was not described. No recommended smartphone-based interventions were identified in this review.

#### Additional Strategies Recommended for Alleviating Caregiver Burden

The additional recommended intervention strategies were financial support, including the provision of a disability grant, fee-free mental health services, medical insurance, and free medication for care recipients [7,57,68]. The recommended social support comprised medical care social security and social resources [52,54,56,57,69]. Recommended counseling interventions comprised basic counseling and supportive psychotherapy [64,69]. Occupation-based interventions included participation in physical and leisure activities [64]. Policy and legislature recommended interventions comprised the implementation of national health insurance schemes, advocacy for a strong mental health policy, integration of caregiver actions and interventions into national mental health care plans, and fee-free mental health services [53,62,68]. Finally, other recommended interventions were access to mental health care, which considers caregiver care inclusion in daily treatment facilities; access to therapeutic tools; availability of mental health units or departments; periodic health checks for caregivers (every 6 months on average); and improved institutional mental health care [35,52,54,67] (Textbox 2).

#### Characteristics of the Implemented Intervention Strategies

Most of the implemented intervention strategies (18/29, 62%) were targeted at alleviating both objective and subjective burden, and 38% (11/29) were exclusively aimed at alleviating subjective burden. The intervention developers were reported as being researchers based on evidence (13/29, 45%) and researchers with expert input (5/29, 17%). In total, 28% (8/29) were existing interventions implemented without any adaptations, and 10% (3/29) of the interventions did not specify the intervention developer. The intervention deliverers identified in the studies included trained peer facilitators (7/29, 24%), researchers (7/29, 24%), psychiatrists and nurses (5/29, 17%), multiple health care professionals (4/29, 14%), and psychologists (3/29, 10%), and 10% (3/29) of the interventions did not specify who delivered them.

The number of sessions offered in the implemented intervention strategies was between 6 and 8 sessions (13/29, 45%), ≤5 sessions (8/29, 28%), and ≥11 sessions (5/29, 17%), and 10% (3/44) of the interventions did not specify the number of sessions. The duration of the implemented interventions in the studies was 6 to 15 weeks (10/29, 34%), ≤5 weeks (7/29, 24%), and ≥16 weeks (4/29, 14%), and 28% (8/29) of the interventions did not specify the duration. Most interventions (9/29, 31%) were delivered once per week, some were offered twice a week (8/29, 28%), and some of the interventions (12/29, 41%) did not specify the frequency of their sessions. The length of the sessions in the implemented interventions ranged between 2 and 2.5 hours (8/29, 28%), 1 to 1.5 hours (6/29, 21%), and ≤1 hour (1/29, 3%), and 48% (14/29) of the interventions did not specify the length of their sessions (Table 5).

**Table 5:** Characteristics of the implemented strategies for alleviating informal caregiver burden (N=29).

Study	Year of publication	Type of intervention	Type of burden targeted by the intervention	Intervention developer	Intervention deliverer	Number of sessions	Duration of intervention	Frequency of sessions per week	Length of the sessions
Ng et al [56]	2018	CoMHIP^a^	Subjective and objective	Not specified	The Integrated Community Centre for Mental Wellness	Not specified	Not specified	Not specified	Not specified
Rai et al [55]	2018	RESHAPE^b^	Subjective and objective burden	Developed as part of WHO^c^ mhGAP^d^ and PRIME^e^	TPO^f^ Nepal, a Nepali nongovernmental organization	5 sessions	4 days	Not specified	Not specified
Rajai et al [45]	2021	Peer- assisted education	Subjective and objective burden	Researchers and approved by 3 faculty members of the Army College of Medical Sciences	Trained peer facilitators	6 sessions	3 weeks	Twice weekly	1 hour
Zhou et al [44]	2020	FLEP^g^	Subjective and objective burden	FLEP: peer-led psychoeducation program developed from the stress and coping model by Pearlin et al [70]	Group facilitators who were peer specialists of experienced caregivers	8 sessions	8 weeks	Once weekly	2 hours
Dewi et al [37]	2019	Family psychoeducation and care decision without pasung	Subjective and objective burden	Researchers	Researchers	3 sessions	3 weeks	Not specified	35-40 minutes
Tabeleão et al [36]	2018	Psychoeducation	Subjective and objective burden	Not specified	10 psychologists	6 sessions	Not specified	Not specified	Not specified
Ntsayagae [40]	2017	Psychoeducation	Subjective and objective burden	Researcher based on existing literature	Researcher	2 sessions	Not specified	Not specified	Not specified
De Souza et al [39]	2016	Psychoeducation	Subjective and objective burden	Psychoeducational intervention for patients with BD^h^ by Colom et al [71] translated and adapted by Dell-Aglio et al [72]	Psychiatrist responsible for specific case	6 sessions	6 to 8 weeks	Twice weekly	Not specified
Kolostoumpis et al [38]	2015	Psychoeducation	Subjective burden	Adapted from the treatment protocol developed by Reinares et al [73] in the Barcelona Bipolar Disorders Program in Spain	Psychiatrist and psychologist	7 sessions	Not specified	Not specified	Not specified
Jessy and Kalaimathy [35]	2014	Psychoeducation	Subjective and objective burden	Researcher based on WHO guidelines for educational interventions and existing literature. Manual translated into Tamil.	Researcher	5 modules	10 weeks	Twice weekly	1 hour
Fallahi Khoshknab et al [41]	2014	Group psychoeducation	Subjective and objective burden	Researchers based on educational program of psychiatric nursing and psychiatric textbooks	Organized based on educational program of psychiatric nursing and psychiatric textbooks (Campbell [74]) and converted to understandable text for patients and families	4 sessions	4 weeks	Once weekly	2 hours
Navidian et al [42]	2012	Psychoeducation	Subjective burden	Researchers based on the families’ needs and the existing literature	Psychiatrist and mental health nurse	4 sessions	4 weeks	Once weekly	2 hours
Sharif et al [75]	2012	Psychoeducation	Subjective and objective burden	Psychiatrist and psychiatric nurse based on the literature and needs of the families	Psychiatrist, nurse, and guest speakers	10 sessions	5 weeks	Not specified	1.5 hours
Chiu et al [43]	2011	FLEP	Subjective burden	Task force consisting of a mental health social worker, a recovered patient with editorial experience, and a caregiver based on available related local educational materials and the NAMI^i^ Family-to-Family program	Trainers who were themselves family members of people with SMI^j^. They received training and a trainer manual.	8 sessions	Not specified	Once weekly	Not specified
Chien et al [48]	2018	Family-led support program	Subjective and objective burden	Family-led mutual support group—contents were based on similar program protocols and the researcher-developed family mutual support groups for psychotic disorders	Family-led mutual support group—co-led by 2 peer family caregivers along with a researcher and rehabilitation nurse	16 sessions	36 weeks	Twice weekly	2 hours
Mentis et al [49]	2015	Support group intervention	Subjective burden	Nongovernmental mental health organization (NGOMH^k^)	Not mentioned	Not specified	Not specified	Not specified	Not specified
Chien et al [48]	2018	Family-led mutual support program	Subjective burden	6 experts on psychiatric rehabilitation (including psychiatrists, clinical psychologists, and nurse specialists)	Peer leader who received training from the researchers and worked closely with a group leader who was a trained psychiatric nurse	14 sessions	39 weeks	Twice weekly	2 hours
Chien and Chan [47]	2013	FPGP^l^	Subjective and objective burden	Content and format were based on previous programs conducted by Li and Arthur [76] in mainland China. Appropriateness of the content was rated by 7 experts, including psychiatrists, psychologists, and nursing specialists.	Peer support—peer leader supported by principal researcher	14 group sessions	39 weeks	Twice weekly	2 hours
Behrouian et al [62]	2021	Emotional regulation	Subjective burden	On the basis of the Dialectical Behavior Therapy Skills Workbook and CBT^m^ principles	Clinical psychologist	8 sessions	8 weeks	Once weekly	Not specified
Behrouian et al [63]	2020	Emotion regulation training	Subjective and objective burden	Sessions were based on previous studies (Gratz and Gunderson [77]). Trainings were based on the Dialectical Behavior Therapy Skills Workbook (McKay et al [78]).	Clinical psychologist	8 sessions	Not specified	Not specified	Not specified
Faghih and Pahlavanzadeh [79]	2019	CBT	Subjective burden	Researchers based on existing literature	Researcher	16 sessions	8 weeks	Twice weekly	1.5 hours
Khosravi et al [65]	2021	Spirituality-based program	Subjective burden	Researcher	Researcher	6 sessions	8 weeks	Twice weekly	1 hour
Chien et al [58]	2020	Manual-guided PBSP^n^	Subjective and objective burden	Developed by McCann et al [80] in Australia, and its Chinese translated version was validated and refined by the research team.	Psychiatric nurse	5 modules	21 weeks	Not specified	Not specified
McCann et al [59]	2015	Guided self-help manual	Subjective and objective burden	The Good Mood Guide developed by Lifeline South Coast NSW^o^, Australia	Researcher via telephone	8 modules	8 weeks	Once weekly	Not specified
Deb et al [67]	2018	Smartphone-based interventions (MHapps^p^)	Subjective and objective burden	Researcher	Psychiatrist	Not specified	Not specified	Not specified	Not specified
Hyun et al [61]	2018	IEP^q^	Subjective burden	Developed by Hyun et al [61] for community-living PMIs^r^ based on the empowerment theories of Kanter [81] and McLean [82]	Mental health professionals who received training with a written structured intervention protocol from the research team	4 sessions	4 weeks	Once weekly	2 hours
Hou et al [60]	2014	MBSR^s^ program	Subjective burden	Not stated but assumed that the researchers modeled the program on the original MBSR by Kabat-Zinn [83]	3 trained instructors with >3 years of experience in MBSR	8 sessions	8 weeks	Once weekly	2 hours, with a CD of 30-45 minutes for home practice
Zoladl et al [66]	2020	Participatory care model	Subjective burden	Researchers	Researchers and staff at the research site	8 sessions	12 weeks	Once weekly	1.5 hours
Ramirez et al [68]	2017	Standard care—included care from general medicine, psychiatry, psychology, family therapy, neuropsychological rehabilitation, and occupational therapy	Subjective and objective burden	Not stated	Medical officer, psychiatrist, psychologist, and occupational therapist	12-18 sessions	10 weeks	Not specified	Not specified

^a^CoMHIP: community mental health early intervention project.

^b^RESHAPE: Reducing Stigma Among Health Care Providers.

^c^WHO: World Health Organization.

^d^mhGAP: Mental Health Gap Action Programme.

^e^PRIME: Programme for Improving Mental Health Care.

^f^TPO: Transcultural Psychosocial Organization.

^g^FLEP: Family Link Education Programme.

^h^BD: bipolar disorder.

^i^NAMI: National Alliance on Mental Illness.

^j^SMI: severe mental illness.

^k^NGOMH: nongovernmental mental health.

^l^FPGP: Family-led Peer Support Group Program.

^m^CBT: cognitive behavioral therapy.

^n^PBSP: problem-solving–based self-learning program.

^o^NSW: New South Wales.

^p^MHapps: mental health apps.

^q^IEP: integrated empowerment program.

^r^PMI: person with mental illness.

^s^MBSR: mindfulness-based stress reduction.

#### Perceived Effectiveness of the Implemented Intervention Strategies

This review outlines the effectiveness of the implemented intervention strategies as reported by the authors who conducted the studies (Table 4). It should be noted that this scoping review included both published and gray literature, and therefore, no critical appraisal of the studies or meta-analyses were conducted. Therefore, care should be taken as these interpretations may be clouded by author bias [84].

The authors expressed the effectiveness of the implemented intervention strategies by highlighting whether the strategy resulted in a positive or negative outcome for the informal caregivers or their care recipients. Overall, the implemented strategies were reported to be effective in reducing the burden of care and improving the quality of life of informal caregivers. Psychoeducation intervention strategy outcomes included improved coping skills, improved quality of life, reduced anxiety and stress, reduced burden of care, reduced psychological strain, improved knowledge and skills in caregiving, enhanced family functioning, enhanced recovery, relapse prevention, and reduced prolonged admissions. Support group outcomes included alleviation of guilt, enhanced help-seeking behavior, improved quality of life, improved knowledge and skills in caregiving, improved psychosocial health, improved family and patient functioning, and decreased demand for mental health services. CBT outcomes included reduction in burden of care, anxiety, and stress and increased resilience of informal caregivers. Guided self-help outcomes included strengthened positive caregiving experiences, reduced negative caregiving experiences, and increased access to support. The reported outcomes for mindfulness and empowerment interventions included empowerment of caregivers, increased self-efficacy, improved quality of life, and decreased depressive symptoms. Collaborative intervention outcomes included reduced burden of care and increased resilience. The standard care outcomes reported included positive outcomes in caregiving, reduction in the burden of care, and increase in social support for caregivers.

Negative outcomes were only reported for the psychoeducation and mindfulness and empowerment intervention strategies. The reported negative outcomes of psychoeducation interventions were no improvement in the burden of care, quality of life, or self-esteem. The reported negative outcomes of mindfulness and empowerment interventions included short-lived improvement in anxiety, and the authors highlighted no improvement after 3 months of follow-up with the participants and no effect on perceived stress, quality of life, and self-compassion, indicating that the effects of the interventions were not sustainable.

## Discussion

### Principal Findings

This scoping review set out to map the literature on strategies for alleviating the burden of informal caregivers of persons with severe and enduring mental health conditions in low- and middle-income countries. The review identified types of strategies, strategy characteristics, and outcomes of the strategies as reported by the authors. The types of strategies identified were categorized as implemented and recommended intervention strategies and included community-based interventions, psychoeducational interventions, support groups, CBT, spirituality-based interventions, guided self-help, smartphone-based interventions, mindfulness and empowerment, collaborative interventions, standard care, financial and social support, counseling, occupation-based interventions, policy and legislature, and access to mental health.

Most of the implemented and recommended intervention studies (33/44, 75%) were conducted in Asian countries and targeted both subjective and objective burdens, and some studies (18/29, 62%) focused exclusively on subjective burden. This shows an increasing research interest in caregiver interventions in mental health research in Asian countries over the last decade. In contrast, the small number of studies from other low- and middle-income countries possibly confirms the limited research on support strategies for alleviating caregiver burden in mental health. Despite consensus on the high levels of burden of informal caregivers of persons with severe and enduring mental health conditions in these countries [56,85-87] and the importance of supporting caregivers, few studies (44/18,086, 0.24%) implemented and recommended intervention strategies for alleviating caregiver burden. This evidence gap raises significant concerns considering the scarcity of mental health care professionals and limited access to quality mental health services in many low- and middle-income countries. As a result, informal caregivers assume a vital role in the support and care of persons with severe and enduring mental health conditions in these regions [88,89]. Having limited strategies for alleviating the burden of informal caregivers may have dire consequences for the management of persons with severe and enduring mental health conditions in these countries.

The implemented and recommended intervention strategies were mainly focused on both male and female family caregivers. These findings are consistent with the literature as families have long been acknowledged as key stakeholders in the care and management of mental disorders [90-93]. Therefore, it is important to ensure that the strategies for alleviating caregiver burden are targeted specifically to this population, particularly in low- and middle-income countries, where occupying this role is often obligatory [94]. The fact that most implemented intervention strategies were aimed at both male and female caregivers is important to note. Although caregiving is identified as a female-oriented role, there is evidence that men also occupy the primary caregiver role and are also likely to experience high levels of caregiver burden [20,94]. This emphasizes the need to ensure that, where strategies for alleviating caregiver burden are implemented, they focus on all caregivers irrespective of their gender. The care recipients in these studies were mostly diagnosed with schizophrenia and mood disorders. This is consistent with the literature highlighting that depressive disorders, schizophrenia, and bipolar disorders are among the top 10 leading causes of disability in low- and middle-income countries [95].

The findings of this scoping review emphasize the need for evidence-based intervention strategies aimed at alleviating the burden of informal caregivers in low- and middle-income countries. Most implemented intervention strategies were informed by evidence, in which researchers consulted existing literature and sought expert input to develop their interventions. Trained peer facilitators delivered most of the implemented interventions and were informal caregivers themselves. This is important to note as it aligns with task shifting, which is focused on transferring skills and responsibilities to local people with the aim of increasing access to mental health services in low- and middle-income countries where there is a shortage of human resources [96,97]. In addition, the findings of this review reveal that researchers, nurses, and psychiatrists offered some of the interventions, which may not be sustainable in low- and middle-income countries given the shortage of mental health professionals [95]. Most studies (13/29, 45%) offered 6 to 8 sessions over 6 to 8 weeks, and the sessions were facilitated once or twice a week for 1 to 2 and a half hours. Given the number and frequency of sessions, it may be useful to use peer facilitators to offer these interventions as most are already in the community, which will ensure sustainability in resource-constrained contexts in low- and middle-income countries.

Overall, the implemented intervention strategies were reported to have a positive effect on alleviating the burden of informal caregivers of people with severe and enduring mental health conditions in low- and middle-income countries. In some studies, these effects were reported for both informal caregivers and their care recipients. The authors reported the effectiveness of the strategies in their studies in terms of the help, benefit, and effect that a specific implemented intervention strategy had on the informal caregivers. Some compared their interventions mainly with standard psychiatric care and psychoeducation, which focused on providing caregivers with information on specific or general mental disorders. Psychoeducational interventions were frequently implemented intervention strategies and were identified as helpful, beneficial, and effective in reducing caregiver burden [35,38,42,75]. This type of intervention was reported to improve caregiver knowledge and skills to enable them to cope with the demands of caregiving. In addition, psychoeducational interventions were linked with positive patient outcomes as they were reported to enhance patient recovery, which subsequently reduced relapses and prolonged admissions. These interventions were commended for being simple, feasible, and cost-effective, making them the most preferred form of intervention strategy to address the burden of informal caregivers.

Similarly, implemented intervention strategies such as support groups, community-based interventions, guided self-help, mindfulness and empowerment, and CBT were reported to have a positive effect on the burden of informal caregivers in low- and middle-income countries. It is important to note that the family-led mutual support program, a peer-facilitated intervention, was reported to have long-term desirable benefits on the psychosocial health of both caregivers and their care recipients compared with the psychoeducational program and standard care offered by psychiatrists, clinical psychologists, and nursing specialists. Similarly, the manual-guided problem-solving–based self-learning program, a guided self-help intervention, was reported to have a superior treatment effect on caregiver burden, care recipients’ symptom severity, and the duration of rehospitalizations at the 6-month follow-up compared with a well-accepted family psychoeducation group program. This implies that support groups and guided self-help, although not frequently implemented, should be considered beneficial strategies for alleviating caregiver burden. In addition, as self-directed and peer-evaluated interventions, these intervention strategies may be considered cost-effective in extending the services to informal caregivers, especially in low- and middle-income countries where a shortage of human resources affects the delivery and quality of mental health services.

Although not frequently implemented, intervention strategies such as spirituality-based, collaborative, and standard care interventions were also reported to reduce caregiver burden. Spirituality-based interventions were identified as an inexpensive and readily available resource for alleviating the burden of informal caregivers. Similarly, the participatory care model was reported to be an efficient and low-cost method for reducing caregiver burden and increasing caregiver resilience. Therefore, it is necessary to further explore the use of these interventions with informal caregivers of people with severe and enduring mental health conditions in low- and middle-income countries.

Despite their potential, the use of virtual interventions or telehealth strategies for informal caregivers remains underrepresented in the literature. This may be attributed to the relatively slow adoption of telehealth among informal caregivers. Notably, only 1 smartphone app was identified in this scoping review. However, telehealth holds promise as a viable approach for disseminating psychoeducational information, providing real-time support, and facilitating participation in virtual caregiver support groups, particularly in remote areas. The increasing proliferation of smartphones and internet access in Africa further emphasizes opportunities for the development of telehealth programs tailored to caregivers of individuals with severe and enduring mental health conditions. Although most of the implemented intervention strategies reported positive effects on caregiver burden, there were negative outcomes reported for some interventions. Hou et al [60] reported that the mindfulness-based stress reduction program led to a short-lived improvement in the stress and anxiety of the informal caregivers but that no improvement was noted after the 3-month follow-up. In addition, this intervention did not demonstrate a sustained effect on health-related quality of life, perceived stress, or self-compassion. These outcomes were attributed to the loss to follow-up and the fact that the measurement instruments could have been less sensitive to changes in quality of life and stress. Similarly, the 6-session individual psychoeducation intervention for informal caregivers of persons with bipolar mood disorder showed no effect on burden, quality of life, or self-esteem. This was attributed to the reduction in the number of sessions from 21 in the original instruction [71] to 6 in this study [35]. Another reason was that the sessions were conducted individually, whereas the literature highlights that multifamily intervention groups are effective [71,73,98].

### Implications for Practice and Policy

The findings of this review provide evidence for the reported evaluated and recommended interventions having benefits in reducing the burden of care among informal caregivers in low- and middle-income countries. Previous studies conducted in low- and middle-income countries have since emphasized the urgent need to strengthen informal caregiver support regarding mental health. The need for information and skills in handling mental health care users, as well as the need for emotional and tangible support, has been highlighted in previous studies [42,99]. The findings of this review revealed that psychoeducation and support groups were highly used strategies for improving knowledge and skills as well as building support for informal caregivers in low- and middle-income countries. Furthermore, these strategies were reported to be beneficial and cost-effective, thereby making them a viable option for implementation in low- and middle-income countries, where limited access to mental health resources prevails [100]. The need to ensure that these strategies are offered on a continuous basis was highlighted in many studies, indicating the need for 6 to 10 sessions over a period of 6 to 10 weeks [7]. To ensure sustainability, training of peer facilitators to deliver these interventions may be realistic as this aligns with a task-shifting approach, which has long been advocated for as a cost-effective strategy for increasing access to mental health services in low- and middle-income countries [101]. It is interesting to note that peer support groups and guided self-help were reported to have long-term benefits on caregiver burden compared with psychoeducation; this highlights that using self-directed interventions is considered a practical option for alleviating informal caregiver burden. In low- and middle-income countries where a shortage of human resources prevails, it is important to consider such interventions as they empower informal caregivers to build support and take ownership of their health and well-being [35]. Spiritual-based interventions, although not frequently implemented, are important to note in the context of low- and middle-income countries as most informal caregivers have been identified as relying on spiritual and religious coping [102,103]. Recommended strategies such as the provision of financial and social support as well as policy and legislature strategies call for an urgent need for policies in mental health to shift focus toward integrating caregiver-oriented services into practice. In addition, these findings highlight the need to adopt an intersectoral approach [104] in which various sectors such as religious and spiritual organizations provide mental health services to extend their accessibility to informal caregivers.

### Limitations

In alignment with Arksey and O’Malley, this review did not include a critical appraisal to ascertain the quality of the studies as the purpose of this scoping review was to map existing strategies for alleviating caregiver burden and report on the outcomes as stated by the authors. In their study, Woo et al [105] cautioned that the exclusion of critical appraisals in scoping reviews means that the review cannot ascertain the research gaps that it aims to address if the included studies are of poor methodological quality. Although this scoping review provides evidence on the existing strategies for alleviating caregiver burden, it is important that the suggested strategies be evaluated in the specific context to ensure their effectiveness before implementation in clinical practice. This step was omitted as the purpose of this review was to map the available intervention strategies and the outcomes reported by the authors in alleviating burden among informal caregivers in low- and middle-income countries. This review only included studies conducted in low- and middle-income countries, and only papers written in English were considered, thus reducing the extent and scope of the evidence on the strategies for alleviating burden among informal caregivers regarding mental health.

### Conclusions

The findings of this scoping review provided the authors with categories they can use to develop semistructured interview guides to use when exploring the existing formal and informal community mental health services to alleviate the burden of informal caregivers in rural South Africa. The categories outline the different types of strategies that can be used to alleviate caregiver burden, in particular the types of strategies offered to informal caregivers and the intervention developers and deliverers in and outside the mental health care system. Although most of the included studies (29/44, 66%) implemented these strategies, a few studies conducted in other low- and middle-income countries (15/44, 34%) recommended the use of these interventions to alleviate caregiver burden. Future studies from low- and middle-income countries in other continents, including Africa and South and North America, should address this gap in the research by evaluating these intervention strategies to alleviate the burden of informal caregivers.

Critical appraisal, which is used to ascertain the quality of the studies, was omitted as the purpose of this scoping review was to map existing strategies for alleviating caregiver burden and report on the outcomes as stated by the authors. Future studies should conduct quality appraisals to establish the effectiveness of these strategies in alleviating the burden of informal caregivers. Peer-facilitated support group interventions, although not frequently implemented, were identified as having long-term benefits compared with frequently implemented interventions such as psychoeducation. It is recommended that future research be directed at implementing and evaluating these interventions to alleviate burden in low- and middle-income countries.

## Appendix

Multimedia Appendix 1 Data extraction templates.

Multimedia Appendix 2 PRISMA-ScR Checklist.

## Abbreviations

CBT: cognitive behavioral therapy
PRISMA: Preferred Reporting Items for Systematic Reviews and Meta-Analyses
PRISMA-ScR: Preferred Reporting Items for Systematic Reviews and Meta-Analyses extension for Scoping Reviews

## Data Availability

All data generated or analyzed during this study are included in this published paper.
