# Supplementary material for: Strategies to Alleviate the Burden Experienced by Informal Caregivers of Persons With Severe Mental Disorders in Low- and Middle-Income Countries: Scoping Review
Source: Interact J Med Res. 2024 Jan 18;13:e48587. doi: 10.2196/48587 (PMC10835589; doi:10.2196/48587)
Supplement: Multimedia Appendix 1 [file ijmr_v13i1e48587_app1.docx]

Table 2: Data extraction table adapted from Peters *et al.*, (2020)

| **Scoping Review Details:** | | | |
| --- | --- | --- | --- |
| Scoping Review Title | | |  |
| Scoping Review Objective/s | | |  |
| Scoping Review question/s | | |  |
| **General information:** | | | |
| Study ID | | |  |
| Study title | | |  |
| Lead Author (surname and initials of the lead author) | | |  |
| **Inclusion/Exclusion Criteria:** | | | |
| Population | | |  |
| Concept | | |  |
| Context (country) | Developing country (incl. Africa, Asia and Latin America and the Caribbean) | |  |
|  | Underdeveloped country | |  |
|  | Mixed countries | |  |
| Context | Rural | |  |
| (type of setting) | Urban | |  |
|  | Peri-urban | |  |
|  | Not specified | |  |
| **Type/s of evidence source:** | | | |
| Journal article | | |  |
| Research report | | |  |
| Guideline document | | |  |
| Opinion piece | | |  |
| Research thesis | | |  |
| **Evidence Source Details and Characteristics:** | | | |
| **Aim/s of the study** | | |  |
| Citation details | Author/s | |  |
|  | Date of publication | |  |
|  | Journal | |  |
|  | Volume/Issue/Article Number | |  |
|  | Pages | |  |
| Country (exact country where the study took place) | | |  |
| Context (specify the setting where the study took place) | | |  |
| Research approach | Quantitative | |  |
|  | Qualitative | |  |
|  | Mixed methods | |  |
|  | Multimethod | |  |
| Study design | Randomised controlled trial | |  |
|  | Non-randomised experimental study | |  |
|  | Cohort study | |  |
|  | Cross-sectional study | |  |
|  | Case control study | |  |
|  | Qualitative research | |  |
|  | Quantitative research | |  |
|  | Prevalence study | |  |
|  | Case series | |  |
|  | Case report | |  |
|  | Clinical prediction rule | |  |
|  | Practice guideline | |  |
|  | Text and opinion | |  |
|  | Other | |  |
| Study funding sources | | |  |
| Possible conflicts of interest for study authors | | |  |
| **Participants details:** | | | |
| Age of caregiver (comment if mixed age) | | |  |
| Age of care recipient (comment if mixed age) | | |  |
| Gender of caregiver | Female | |  |
|  | Male | |  |
|  | Mixed gender groups (male and female) | |  |
| Gender of care recipient | Female | |  |
|  | Male | |  |
|  | Mixed gender groups (male and female) | |  |
| Diagnosis of the care recipient | | |  |
| Sampling size | | |  |
| Total number of participants | | |  |
| **Intervention type:** | | | |
| Intervention content | Type of intervention | |  |
|  | Who developed the intervention | |  |
|  | Who delivered the intervention | |  |
|  | Type of burden targeted by the intervention (objective, subjective etc.) | |  |
| Intervention description | | Duration of intervention |  |
|  |  | Number of sessions (overall) |  |
|  |  | Number of sessions (over weeks) |  |
|  |  | Location for intervention |  |
|  |  | Measurement instruments |  |
| **Conclusion and recommendations extracted from source of evidence:** | | | |
| Conclusions | | |  |
| Recommendations | | |  |

Table 3: Data extraction table adapted from Peters *et al.*, (2020)

| **Scoping Review Details:** | | | | |
| --- | --- | --- | --- | --- |
| Scoping Review Title | | | |  |
| Scoping Review Objective/s | | | |  |
| Scoping Review question/s | | | |  |
| **General information:** | | | | |
| Study ID | | | |  |
| Study title | | | |  |
| Lead Author (*surname and initials of the lead author)* | | | |  |
| **Inclusion/Exclusion Criteria:** | | | | |
| Population | | | |  |
| Concept | | | |  |
| Context (country) | | Developing country (incl. Africa, Asia and Latin America and the Caribbean) | |  |
|  |  | Underdeveloped country | |  |
|  |  | Mixed countries | |  |
| Context | | Rural | |  |
| (type of setting) | | Urban | |  |
|  | | Peri-urban | |  |
|  | | Not specified | |  |
| **Type/s of evidence source:** | | | | |
| Public health policy | | | |  |
| Mental health policy | | | |  |
| Guideline document | | | |  |
| **Evidence Source Details and Characteristics:** | | | | |
| **Aim/s of the policy document** | | | |  |
| **Objective/s of the policy document** | | | |  |
| Citation details | Author/s | | |  |
|  | Date of publication | | |  |
|  | Pages | | |  |
| Country | | | |  |
| Context (urban/peri-urban/rural) | | | |  |
| **Target population:** | | | | |
| Age of caregiver (comment if mixed age) | | | |  |
| Age of care recipient (comment if mixed age) | | | |  |
| Gender of caregiver | | Female | |  |
|  |  | Male | |  |
|  |  | Mixed gender groups (male and female) | |  |
| Gender of care recipient | | Female | |  |
|  |  | Male | |  |
|  |  | Mixed gender groups (male and female) | |  |
| Population (*description of the population that the policy is targeted towards)* | | | |  |
| **Characteristics of policy document:** | | | | |
| Execution plan | | | |  |
| Defined procedure | | | |  |
| Periodical review | | | |  |
| **Intervention type:** | | | | |
| Intervention content | | Type of intervention | |  |
|  |  | Who developed the intervention | |  |
|  |  | Who delivered the intervention | |  |
|  |  | Type of burden targeted by the intervention (objective, subjective etc) | |  |
| Intervention description | | | Duration of intervention |  |
|  |  |  | Number of sessions (overall) |  |
|  |  |  | Number of sessions (over weeks) |  |
|  |  |  | Location for intervention |  |
|  |  |  | Measurement instruments |  |
| Targeted output | | | |  |
| **Recommendations extracted from source of evidence:** | | | | |
| Conclusions | | | |  |
| Recommendations | | | |  |
